# Reduced Histone H3 Lysine 9 Methylation Contributes to the Pathogenesis of Latent Autoimmune Diabetes in Adults via Regulation of SUV39H2 and KDM4C

**DOI:** 10.1155/2017/8365762

**Published:** 2017-03-15

**Authors:** Xi-yu Liu, Hong Li

**Affiliations:** ^1^Department of Endocrinology, Fourth Affiliated Hospital of Zhejiang University School of Medicine, Yiwu, Zhejiang, China; ^2^Department of Endocrinology, Sir Run Run Shaw Hospital, School of Medicine, Zhejiang University, Hangzhou, Zhejiang, China

## Abstract

*Aims*. Latent autoimmune diabetes in adults (LADA) is an autoimmune disease of which the mechanism is not clear. Emerging evidence suggests that histone methylation contributes to autoimmunity.* Methods*. Blood CD4^+^ T lymphocytes from 26 LADA patients and 26 healthy controls were isolated to detect histone H3 lysine 4 and H3 lysine 9 methylation status.* Results*. Reduced global H3 lysine 9 methylation was observed in LADA patients' CD4^+^ T lymphocytes, compared to healthy controls (*P* < 0.05). H3 lysine 4 methylation was not statistically different. The reduced H3 lysine 9 methylation was associated with GADA titer but not correlated with glycosylated hemoglobin (HbA1c). When the LADA patient group was divided into those with complication and those without, relatively reduced global H3 lysine 9 methylation was observed in LADA patients with complication (*P* < 0.05). The expression of histone methyltransferase SUV39H2 for H3 lysine 9 methylation was downregulated in LADA patients, and the expression of histone demethylase KDM4C which made H3 lysine 9 demethylation was upregulated.* Conclusion*. The reduction of histone H3 lysine 9 methylation which may due to the downregulation of methyltransferase SUV39H2 and the upregulation of demethylase KDM4C was found in CD4^+^ T lymphocytes of LADA patients.

## 1. Introduction

The prevalence of diabetes in Chinese adults was estimated to be 11.6% according to a survey in 2013 [1]. Latent autoimmune diabetes in adults (LADA) is a subtype of type 1 diabetes. The total prevalence of LADA was as high as 8.63% and GADA positive LADA was 5.9% in clinical newly diagnosed type 2 diabetes in China [2], so the number of Chinese LADA patients was large. LADA is an autoimmune disease characterized by T-cell-mediated immune destruction of the islet *β*-cells, and CD4^+^ T lymphocytes play a central role in the pathogenesis of LADA [3–6]. Although extensive evidence showed that LADA was an autoimmune disease, the exact molecular mechanism remains to be explored [7].

In the recent decades, histone methylation, which helped the genome expression, has become a research hotspot. Evidence has demonstrated that histone methylation played an important role in autoimmune diseases [8, 9]. Recent research also showed that histone methylation played an important role in the pathogenesis of diabetes [10, 11]. LADA was an autoimmune disease, so we hypothesized that histone methylation may associate with the pathogenesis of LADA.

Miao et al. studied histone modification in classical type 1 diabetes patients. They found that lymphocytes from classical type 1 diabetes patients displayed distinct profile of chromatin histone 3 lysine 9 dimethylation [12], and H3 lysine 9 acetylation levels were markedly varied in classical type 1 diabetes monocytes relative to controls [13].

Type 1 diabetes is divided into two subtypes (classical type 1 diabetes and LADA) [14, 15]. The present study of histone methylation in type 1 diabetes patients focused on classical type 1 diabetes, rarely in LADA. Therefore, it is important to perform research of histone methylation in LADA. The present study of histone methylation in type 1 diabetes chose lymphocytes or monocytes, but CD4^+^ T lymphocytes play a central role in the pathogenesis of LADA. Therefore, we investigated the global histone methylation pattern and the gene expression status of specific histone methyltransferases and demethylases in CD4^+^ T lymphocytes from LADA patients and healthy controls.

## 2. Methods

### 2.1. Patients and Controls

**Figure 1:**
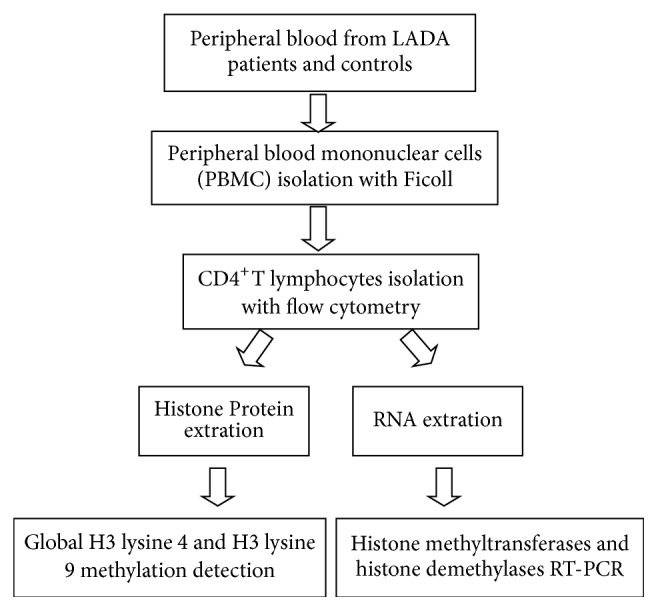
The study scheme. Twenty-six LADA patients and 26 healthy control subjects were enrolled in this study. Peripheral blood mononuclear cells (PBMC) were isolated with Ficoll from peripheral venous blood which was obtained from each participant. CD4^+^ T lymphocytes were isolated by flow cytometry. Then, Global H3 lysine 4 and H3 lysine 9 methylation was detected in CD4^+^ T lymphocytes from 26 subjects. The mRNA level of histone methyltransferases gene (SET1, SUV39H1, SUV39H2, and G9a) and histone demethylases gene (LSD1, KDM3A, KDM4A, KDM4B, KDM4C, KDM5A, KDM5B, KDM5C, and KDM5D) was detected by real-time PCR.

We enrolled 52 volunteers with 26 LADA patients and 26 healthy controls. Patients who were considered to have had LADA with age above 30 years old on the basis of glutamic acid decarboxylase autoantibody (GADA) positive were eligible to undergo this study if they had no history of tumor, other immune disease. The controls were considered to be healthy on the basis of normal oral glucose tolerance test (OGTT) and negative GADA if they had no history of immune disease, diabetes, or tumors. The LADA patients of this study included 12 patients absent of diabetic complication and 14 patients with at least one microvascular or macrovascular complication, specifically, 8 patients with retinopathy, 3 patients with neuropathy, 6 patients with nephropathy, and 4 patients with macrovascular complication. GADA positivity above or below 180 units/ml was the accepted threshold to divide LADA into two groups, with high GADA titer (≥180 units/ml) and low GADA titer (<180 units/ml and ≥18 units/ml). Experimental scheme of this study was shown in Figure 1. Our research was in compliance with the Helsinki Declaration. The ethics approval and consent were approved by the Ethics Committee of Sir Run Run Shaw Hospital, School of Medicine, Zhejiang University, and the Fourth Affiliated Hospital of Zhejiang University School of Medicine.

### 2.2. Cells Preparation

Peripheral venous blood was obtained from each patient and healthy control. Then, peripheral blood mononuclear cells were isolated by density gradient centrifugation using Ficoll gradients. CD4^+^ T lymphocytes were separated by flow cytometry and stored at −80°C. The antibodies used for cell sorting in the flow cytometry were as follows: FITC mouse anti-human CD3 clone antibodies and FITC mouse IgG1, *κ* isotype control, APC-H7 mouse anti-human CD4 clone antibodies and APC-H7 mouse IgG1, *κ* isotype control (BD Company, America).

### 2.3. Detection of Global H3 Lysine 4 and H3 Lysine 9 Methylation

Global H3 lysine 4 and H3 lysine 9 methylation were detected according to the manufacturer's instructions of the EpiQuik™ global histone H3 lysine 4 and H3 lysine 9 methylation assay kits (Epigentek, America). Cells were collected into a tube and the lysis buffer was used to lyse cells. Histone was extracted by using extraction buffer and TCA solution. Histone proteins of samples and the methylated H3 lysine 4 or H3 lysine 9 control were spotted on the strip wells. Capture antibody was added after wash. The methylated histone H3 lysine 4 or H3 lysine 9 was recognized with high-affinity antibody. The histone H3 lysine 4 or H3 lysine 9 methylation was quantified by calculating OD (sample-blank)/OD (untreated control-blank).

### 2.4. Real-Time PCR

Real-time PCR was performed using the one-step SYBR PrimeScript™ RT-PCR kit according to the manufacturer's instructions (Takara, Japan). *β*-Actin was used as an internal control to normalize for differences in the amount of total RNA of each sample. The histone methyltransferases (SET1, G9a, SUV39H1, and SUV39H2) and the histone demethylases (LSD1, KDM3A, KDM4A, KDM4B, KDM4C, KDM5A, KDM5B, KDM5C, and KDM5D) gene expression was detected by real-time quantitative PCR. Primer sequences of the above histone methylation modifier genes were listed in Table 1.

### 2.5. Statistical Analysis

Results were expressed as the mean ± SD and analyzed using Student's* t*-test for continuous variables. Correlation was determined by Pearson's rank order correlation or Spearman's rank order correlation, depending on the normality of the data. *P* < 0.05 were considered as significant. Real-time PCR data was analyzed by the comparative CT method (also named the 2^−ΔΔCT^ method). The absolute of fold change > 2 was considered as significant.

## 3. Results

### 3.1. Characteristics of the Patients and Controls

The characteristics of the patients and healthy controls were shown in Table 2. There were no statistical differences in age, sex proportion, BMI, total cholesterol, triglyceride, HDL cholesterol, LDL cholesterol, and serum creatinine between LADA patients group and healthy controls group (*P* > 0.05), but there were significant differences in HbA1c and diabetes duration between the two groups (*P* < 0.05). LADA group contained 12 patients without any diabetes complication and 14 patients with diabetes complications. When the LADA group was divided into those with complications and those without complication, there was statistical difference in HbA1c and diabetes duration between two subgroups (*P* < 0.05).

### 3.2. Reduced H3 Lysine 9 Methylation in LADA Patients

**Figure 2:**
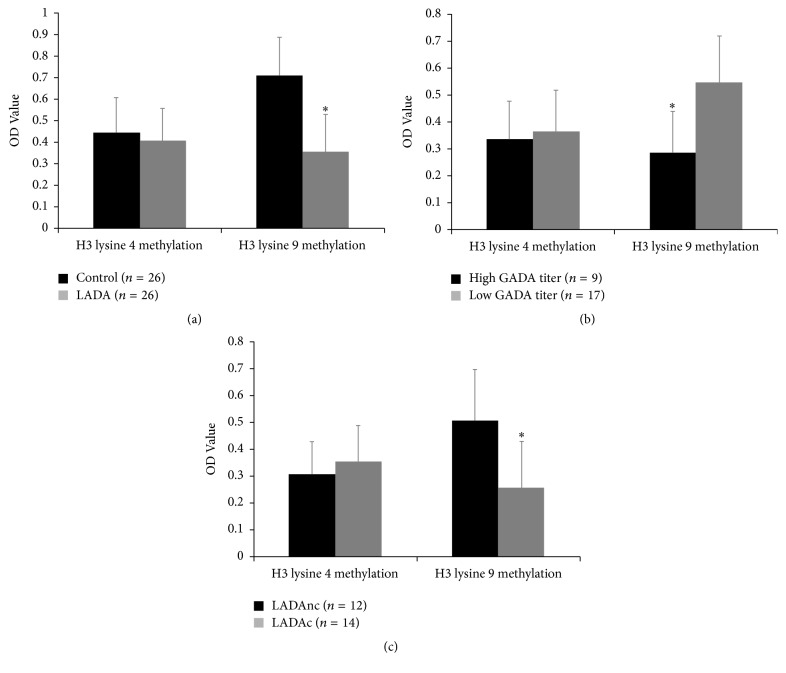
Reduced H3 lysine 9 methylation level in LADA patients. (a) Global H3 lysine 4 and H3 lysine 9 methylation level in CD4^+^ T lymphocytes from 26 LADA patients and 26 healthy controls, ^*∗*^*P* < 0.05, LADA patients versus healthy controls. (b) Global H3 lysine 4 and H3 lysine 9 methylation level in CD4^+^ T lymphocytes from LADA patients, ^*∗*^*P* < 0.05, LADA patients with low GADA titer (*n* = 17) versus LADA patients with high GADA titer (*n* = 9). (c) Global H3 lysine 4 and H3 lysine 9 methylation level in in CD4^+^ T lymphocytes from LADA patients, ^*∗*^*P* < 0.05, LADA patients with complications (LADAc, *n* = 14) versus LADA patients without complications (LADAnc, *n* = 12).

We separated CD4^+^ T lymphocytes from 26 LADA patients and 26 healthy controls to investigate global histone H3 lysine 4 and H3 lysine 9 methylation levels. Reduced global H3 lysine 9 methylation was observed in CD4^+^ T lymphocytes of LADA patients compared to healthy controls (*P* < 0.05). However, H3 lysine 4 methylation level showed no statistical difference between the two groups (Figure 2(a)).

### 3.3. The Association between H3 Lysine 9 Methylation and GADA Titer

LADA was an autoimmune disease, and GADA positive was used to classify LADA. Every LADA patient in this study was GADA positive. We then compared the H3 lysine 9 methylation level between LADA patients with low GADA titer (18 units/ml ⩽ GADA < 180 units/ml) and LADA patients with high GADA titer (GADA *⩾* 180 units/ml). We found that reduced global H3 lysine 9 methylation level was observed in CD4^+^ T lymphocytes of LADA patients with high GADA titer (*P* < 0.05) (Figure 2(b)).

### 3.4. H3 Lysine 9 Methylation Was Not Significantly Correlated with HbA1c

We then analyzed the association between H3 lysine 9 methylation and clinical characteristics. We found that LADA CD4^+^ T lymphocytes H3 lysine 9 methylation was not related to HbA1c, gender, age, BMI, diabetes duration, fasting C-peptide, total cholesterol, triglyceride, HDL cholesterol, LDL-C cholesterol, and serum creatinine (*P* > 0.05), as seen in Table 3.

### 3.5. H3 Lysine 9 Methylation Associated with Complications of Diabetes

In order to assess whether H3 lysine 9 methylation was associated with complications of diabetes. We divided the LADA patients group into two subgroups, those with complications (*n* = 14) and those without complication (*n* = 12). We found that relatively reduced global H3 lysine 9 methylation (*P* < 0.05) was observed in CD4^+^ T lymphocytes of LADA patients with complications compared to those without complication (Figure 2(c)).

### 3.6. Gene Expression of Histone Methyltransferases and Demethylases

In order to investigate the cause of altered histone methylation patterns in LADA patients, we assessed mRNA levels of histone methyltransferases (SET1, G9a, SUV39H1, and SUV39H2) and the histone demethylases (LSD1, KDM3A, KDM4A, KDM4B, KDM4C, KDM5A, KDM5B, KDM5C, and KDM5D) in CD4^+^ T lymphocytes by real-time quantitative PCR.

**Figure 3:**
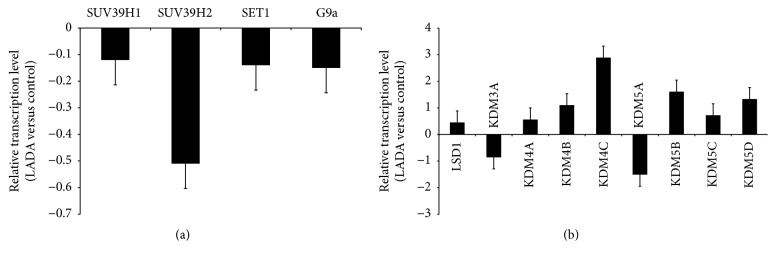
The mRNA level of histone methyltransferase and demethylase in CD4^+^ T lymphocytes from LADA patients. Data are normalized against beta-actin. The ΔCt values were from 0.5 to 3.9. (a) Relative mRNA levels of histone methyltransferases (SET1, G9a, SUV39H1, and SUV39H2) in CD4^+^ T lymphocytes from LADA patients (*n* = 26) and healthy controls (*n* = 26), as measured by real-time PCR. The absolute of fold change > 2 was considered significant, LADA patients versus healthy controls; data are normalized against beta-actin. (b) Relative mRNA levels of histone demethylases (LSD1, KDM3A, KDM4A, KDM4B, KDM4C, KDM5A, KDM5B, KDM5C, and KDM5D) in CD4^+^ T lymphocytes from LADA patients (*n* = 26) and healthy controls (*n* = 26), the absolute of fold change > 2 was considered significant, LADA patients versus healthy controls; data are normalized against beta-actin.

**Figure 4:**
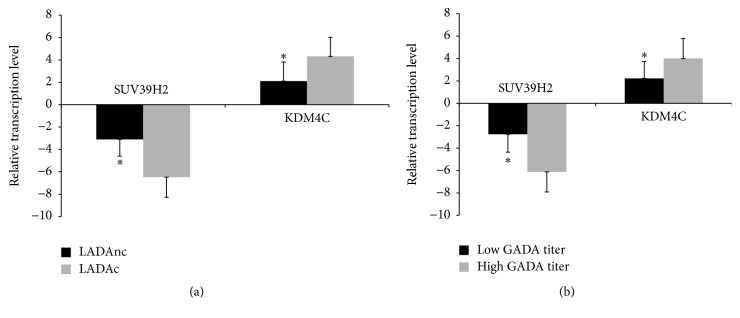
An association of complication and GADA titer with the expression of SUV39H2 and KDM4C in CD4^+^ T lymphocytes from LADA patients was visible. (a) Relative mRNA levels of histone methyltransferase SUV39H2 and demethylase KDM4C in CD4^+^ T lymphocytes from LADA patients, ^*∗*^*P* < 0.05, LADA patients with complications (LADAc, *n* = 14) versus LADA patients without complications (LADAnc, *n* = 12). (b) Relative mRNA levels of histone methyltransferase SUV39H2 and demethylase KDM4C in CD4^+^ T lymphocytes from LADA patients, ^*∗*^*P* < 0.05, LADA patients with low GADA titer (*n* = 17) versus LADA patients with high GADA titer (*n* = 9).

As shown in Figure 3, we found that the expression of histone methyltransferase SUV39H2 in LADA patients was downregulated, respectively, compared to healthy controls (Figure 3(a)). The expression of histone demethylase KDM4C was upregulated in LADA patients compared to healthy controls (Figure 3(b)). Other histone methyltransferases and demethylases detected in our study showed no statistical difference between LADA patients and controls. Compared to LADA patients without complication, the gene expression of SUV39H2 was downregulated and KDM4C was upregulated in LADA patients with complication (*P* < 0.05, Figure 4(a)). In the meantime, compared to LADA patients with low GADA titer, the gene expression of SUV39H2 was downregulated and KDM4C was upregulated in LADA patients with high GADA titer (*P* < 0.05, Figure 4(b)).

## 4. Discussion

The prevalence of diabetes is increasing year by year. LADA is a subtype of diabetes of which exact pathogenesis is unknown. Histone methylation is a dynamic epigenetic process that participates in a diverse array of cellular processes and has been found to associate with autoimmune diseases, including type 1 diabetes [9–12]. The objective of this study was to explore whether histone methylation was associated with the pathogenesis of LADA. We chose CD4^+^ T lymphocytes to study, and the reason was as follows. Firstly, these cells can be separated from peripheral blood which could be obtained during a regular follow-up. Next and more importantly, although there were many immune cells involved in the pathogenesis of LADA, including CD4^+^ T lymphocytes, CD8^+^ T lymphocytes, B lymphocytes, and monocytes, CD4^+^ T lymphocytes play a central role in the pathogenesis of LADA [3–6].

We demonstrated the global histone 3 lysine 4 and lysine 9 methylation pattern in CD4^+^ T lymphocytes from LADA patients. We found that reduced global H3 lysine 9 methylation existed in CD4^+^ T lymphocytes from LADA patients, and the reduced H3 lysine 9 methylation was associated with GADA titer but not significantly correlated with blood glucose (HbA1c). Glutamic acid decarboxylase (GAD) has been recognized as a target antigen in type 1 diabetes. Treatment with GAD in the nonobese diabetes mouse, a model of type 1 diabetes, can prevent diabetes. GAD formulated with aluminum (alum), using in a dose-finding study in LADA, would preserve insulin secretion. GAD autoantibodies (GADA) is undoubtedly one of conditions for a confirmatory LADA diagnosis. In fact, GADA is far from being solely a biomarker but has its specific role in the pathogenesis of LADA. As H3 lysine 9 methylation was correlated with GADA, we speculated that H3 lysine 9 methylation may contribute to the pathogenesis of LADA.

Histone methylation, which was regulated by specific histone methyltransferases and histone demethylases, affects chromatin compaction and transcription. Histone methyltransferases and demethylases were promising targets for new drugs for autoimmune diseases [16–19]. Histone 3 lysine 4 methylation was regulated by histone methyltransferase SET1 and histone demethylase LSD1, KDM5A, KDM5B, KDM5C, and KDM5D. Histone 3 lysine 9 methylation was regulated by histone methyltransferase G9a, SUV39H1, SUV39H2, and histone demethylase LSD1, KDM3A, KDM3B, KDM4A, KDM4B, and KDM4C [20, 21]. In this study, we found that the expression of histone methyltransferase SUV39H2 in LADA patients was downregulated and the expression of histone demethylase KDM4C was upregulated. SUV39H2 and KDM4C were responsible for histone 3 lysine 9 methylation and demethylation. We speculated that the low expression of the SUV39H2 and high expression of KDM4C may explain the reduction of global histone H3 lysine 9 methylation in the CD4^+^ T lymphocytes from LADA patients.

We further studied the association of H3 lysine 9 methylation with complications of diabetes. The results showed that LADA patients with complications had a relatively reduced global H3 lysine 9 methylation compared to LADA patients without complication. HbA1c was related to the progression of complications [22, 23]. We found H3 lysine 9 methylation was correlated with complications of diabetes but not correlated with HbA1c. We speculated that H3 lysine 9 methylation was an independent factor for the progression of complications of LADA.

Islet transplantation was shown to have positive consequences on diabetic complications [24]. In kidney-pancreas transplanted patients, most of metabolic and functional features of the central nervous system appeared to be near normalized after a 5-year follow-up period of sustained normoglycemia [25]. Possibly, histone H3 lysine 9 methylation lever would be normalized after islet transplantation in LADA patients. It would be valuable to perform such research.

In conclusion, we found a reduced histone H3 lysine 9 methylation pattern in LADA with low expression of the methyltransferase SUV39H2 and high expression of the demethylase KDM4C.

## Figures and Tables

**Table 1:** Primer sequences used in real-time PCR.

Gene	Forward primer (5′-3′)	Reverse primer (5′-3′)
SET1	GACACAAGCTTCTCCAGCA	TGAAGATGCAGAGAAGTGGC
SUV39H1	AAGAAGATCCGCGAACAGGA	GGAAGTGCTTGAGGATACGGAC
SUV39H2 G9a	ATCCCACCTGGTACTCCCATCT ACAAAGAGGGGGACACAGCA	GCAAAGCGAATACTGTGTGCC ATGGTGGACGTCTCGCAGTT
LSD1	TTCTGGAGGGTATGGAGACG	ACCTTCTGGGTCTGTTGTGG
KDM3A	GTGCTCACGCTCGGAGAAA	AAACAGCTCGAATGGTCCCG
KDM4A	AGAGTTCCGCAAGATAGCCAA	AGTCCAGGATTGTTCTCAGCC
KDM4B	CTTTGGGGAGCCTAAGTCCTG	GGTAGCCGTAGGGAAATGTGA
KDM4C	GAGGAGCTAGAGGCCAAGC	CTTCCAGGTCGGACCCTTC
KDM5A	CCGTCTTTGAGCCGAGTTG	GGACTCTTGGAGTGAAACGAAA
KDM5B	AAGAGACGCCTGGAAAGAGAG	CCAAACCTCACTGACCTCATC
KDM5C	GGGTCCGACGATTTCCTACC	GCGATGGGCCTGATTTTCG
KDM5D	GGGTCCGACGATTTCCTACC	GCGATGGGCCTGATTTTCG
*β*-ACTIN	GCACCA CAC CTT CTA CAA TGA GC	GGA TAG CACAGC CTG GATAGCAAC

**Table 2:** Characteristics of the participates.

Parameters	LADA patients (*n* = 26)	Control subjects (*n* = 26)	LADA patients	
			Complication-free (*n* = 12)	With complication (*n* = 14)
Gender (male%)	46.2%	46.2%	50.0%	42.9%
Age (years)	43 ± 9	39 ± 9	37 ± 7	48 ± 10
BMI (kg/m^2^)	23.9 ± 3.6	23.6 ± 3.9	23.7 ± 4.1	24.0 ± 4.2
Diabetes duration (years)	9 ± 7	0^*∗∗*^	5 ± 5	12 ± 8^#^
HbA1c (%)	9.6 ± 2.6	5.2 ± 0.3^*∗*^	8.0 ± 2.3	12.1 ± 2.8^#^
FCP (nmol/l)	0.49 ± 0.27	—	0.62 ± 0.32	0.37 ± 0.28
Low FCP	8/26 (30.8%)	—	2/12 (16.7%)	6/14 (42.9%)
High titer GADA	9/26 (34.6%)	—	4/12 (33.3%)	5/14 (35.7%)
Low titer GADA	17/26 (65.4%)	—	8/12 (66.7%)	9/14 (64.3%)
Total cholesterol (mmol/l)	4.2 ± 1.0	4.3 ± 1.0	4.1 ± 0.9	4.3 ± 1.1
Triglyceride (mmol/l)	1.8 ± 1.1	1.9 ± 1.2	1.7 ± 0.9	1.9 ± 1.4
HDL cholesterol (mmol/l)	1.3 ± 0.3	1.2 ± 0.3	1.3 ± 0.3	1.3 ± 0.2
LDL cholesterol (mmol/l)	2.5 ± 0.7	2.4 ± 0.8	2.4 ± 0.7	2.5 ± 0.8
Serum creatinine (umol/l)	102 ± 18.9	80 ± 17.2	96 ± 12.8	108 ± 16.7

*n*, number of subjects; BMI, body mass index; HbA1c, glycated hemoglobin; FCP, fasting C-peptide; HDL, high-density lipoprotein; LDL, low-density lipoprotein; GADA, glutamic acid decarboxylase antibody.

^*∗*^ *P* < 0.05, ^*∗∗*^*P* < 0.01, LADA patients compared with controls.

^#^ *P* < 0.05, LADA patients with complications compared with LADA patients without complications.

**Table 3:** Correlation analysis of global histone H3 lysine 4 and H3 lysine 9 methylation in CD4^+^ T lymphocytes from LADA patients.

	H3 lysine 4 methylation (*n* = 26)		H3 lysine 9 methylation (*n* = 26)	
	*r*	*P*	*r*	*P*
Age	0.456	0.367	0.376	0.235
BMI	0.075	0.789	0.127	0.665
HbA1c	−0.347	0.129	−0.546	0.098
FCP	0.163	0.102	0.174	0.526
Duration of Diabetes	0.223	0.424	0.114	0.197
Total cholesterol	0.015	0.680	−0.186	0.214
Triglyceride	0.074	0.823	−0.235	0.556
HDL cholesterol	0.128	0.276	−0.469	0.109
LDL cholesterol	0.217	0.289	−0.159	0.245
Serum creatinine	−0.136	0.212	−0.267	0.334
GADA titer	−0.336	0.183	−0.824	0.021

H3 lysine 4 methylation and H3 lysine 9 methylation in LADA patients' CD4^+^ T lymphocytes were not correlated with age, BMI, HbA1c, FCP, duration of diabetes, total cholesterol, triglyceride, HDL cholesterol, LDL cholesterol, and serum creatinine. H3 lysine 9 methylation was associated with GADA titer.

*n*, number of subjects; BMI, body mass index; HbA1c, glycated hemoglobin; FCP, fasting C-peptide; HDL, high-density lipoprotein; LDL, low-density lipoprotein; GADA, glutamic acid decarboxylase antibody.

## References

[1] Xu Y., Wang L., He J. (2013). Prevalence and control of diabetes in Chinese adults. *The Journal of the American Medical Association*.

[2] Zhou Z., Xiang Y., Ji L. (2013). Frequency, immunogenetics, and clinical characteristics of latent autoimmune diabetes in China (LADA China Study): a nationwide, multicenter, clinic-based cross-sectional study. *Diabetes*.

[3] Yang Z., Zhou Z., Huang G. (2007). The CD4+ regulatory T-cells is decreased in adults with latent autoimmune diabetes. *Diabetes Research and Clinical Practice*.

[4] Radenkovic M., Silver C., Arvastsson J. (2016). Altered regulatory T cell phenotype in latent autoimmune diabetes of the adults (LADA). *Clinical & Experimental Immunology*.

[5] Marek-Trzonkowska N., Myśliwiec M., Dobyszuk A. (2014). Therapy of type 1 diabetes with CD4+CD25highCD127-regulatory T cells prolongs survival of pancreatic islets—results of one year follow-up. *Clinical Immunology*.

[6] Dahan R., Gebe J. A., Preisinger A. (2013). Antigen-specific immunomodulation for type 1 diabetes by novel recombinant antibodies directed against diabetes-associates auto-reactive T cell epitope. *Journal of Autoimmunity*.

[7] Laugesen E., Østergaard J. A., Leslie R. D. G. (2015). Latent autoimmune diabetes of the adult: current knowledge and uncertainty. *Diabetic Medicine*.

[8] Gupta B., Hawkins R. D. (2015). Epigenomics of autoimmune diseases. *Immunology and Cell Biology*.

[9] Haery L., Thompson R. C., Gilmore T. D. (2015). Histone acetyltransferases and histone deacetylases in B- and T-cell development, physiology and malignancy. *Genes and Cancer*.

[10] Li X., Li C., Li X. (2016). Involvement of histone lysine methylation in p21 gene expression in rat kidney in vivo and rat mesangial cells in vitro under diabetic conditions. *Journal of Diabetes Research*.

[11] Reddy M. A., Zhang E., Natarajan R. (2015). Epigenetic mechanisms in diabetic complications and metabolic memory. *Diabetologia*.

[12] Miao F., Smith D. D., Zhang L., Min A., Feng W., Natarajan R. (2008). Lymphocytes from patients with type 1 diabetes display a distinct profile of chromatin histone H3 lysine 9 dimethylation: an epigenetic study in diabetes. *Diabetes*.

[13] Miao F., Chen Z., Zhang L. (2012). Profiles of epigenetic histone post-translational modifications at type 1 diabetes susceptible genes. *Journal of Biological Chemistry*.

[14] Hernandez M., Mollo A., Marsal J. R. (2015). Insulin secretion in patients with latent autoimmune diabetes (LADA): half way between type 1 and type 2 diabetes: Action LADA 9. *BMC Endocrine Disorders*.

[15] Brophy S., Davies H., Mannan S., Brunt H., Williams R. (2011). Interventions for latent autoimmune diabetes (LADA) in adults. *Cochrane Database of Systematic Reviews*.

[16] Lee J.-Y., Lee S.-H., Heo S.-H. (2015). Novel function of lysine methyltransferase G9a in the regulation of Sox2 protein stability. *PLoS ONE*.

[17] Zhao E., Ding J., Xia Y. (2016). KDM4C and ATF4 cooperate in transcriptional control of amino acid metabolism. *Cell Reports*.

[18] Morera L., Lübbert M., Jung M. (2016). Targeting histone methyltransferases and demethylases in clinical trials for cancer therapy. *Clinical Epigenetics*.

[19] Hui C., Ye T. (2015). Synthesis of lysine methyltransferase inhibitors. *Frontiers in Chemistry*.

[20] Krishnan S., Horowitz S., Trievel R. C. (2011). Structure and function of histone H3 lysine 9 methyltransferases and demethylases. *ChemBioChem*.

[21] Michalak E. M., Visvader J. E. (2016). Dysregulation of histone methyltransferases in breast cancer—opportunities for new targeted therapies?. *Molecular Oncology*.

[22] Genuth S. (2006). Insights from the diabetes control and complications trial/epidemiology of diabetes interventions and complications study on the use of intensive glycemic treatment to reduce the risk of complications of type 1 diabetes. *Endocrine Practice*.

[23] Lachin J. M., White N. H., Hainsworth D. P., Sun W., Cleary P. A., Nathan D. M. (2015). Effect of intensive Diabetes therapy on the progression of diabetic retinopathy in patients with type 1 diabetes: 18 years of follow-up in the DCCT/EDIC. *Diabetes*.

[24] Bassi R., Fiorina P. (2011). Impact of islet transplantation on diabetes complications and quality of life. *Current Diabetes Reports*.

[25] Fiorina P., Vezzulli P., Bassi R. (2012). Near normalization of metabolic and functional features of the central nervous system in type 1 diabetic patients with end-stage renal disease after kidney-pancreas transplantation. *Diabetes Care*.

